# Large cardiac fibroma and teratoma in children- case reports

**DOI:** 10.1186/s13019-015-0242-9

**Published:** 2015-03-22

**Authors:** Neerod Kumar Jha, Laszlo Kiraly, Csaba Tamas, Haitham Talo, Mohammad Daud Khan, Hazem El Badaoui, Anurag Jain, Azzam Hammad

**Affiliations:** 1 Division of Paediatric Cardiac Surgery, Sheikh Khalifa Medical City (managed by Cleveland Clinic), PO Box-51900, Karamah Street, Abu Dhabi, United Arab Emirates; 2 Paediatric Cardiology, Sheikh Khalifa Medical City (managed by Cleveland Clinic), PO Box-51900, Karamah Street, Abu Dhabi, United Arab Emirates; 3 Radiology, Sheikh Khalifa Medical City (managed by Cleveland Clinic), PO Box-51900, Karamah Street, Abu Dhabi, United Arab Emirates; 4 Pathology, Sheikh Khalifa Medical City (managed by Cleveland Clinic), PO Box-51900, Karamah Street, Abu Dhabi, United Arab Emirates

**Keywords:** Cardiac, Surgery, Tumour, Fibroma, Teratoma

## Abstract

Primary cardiac tumours in paediatric population are an unusual occurrence. Although, majority of such tumours are benign (90%), the frequency and type of cardiac tumours in this age group is different from the adult population. There are several consecutive series published in the last decade on cardiac neoplasms. Therefore, this is not only an effort to contribute to the existing literature for better understanding and management of similar patients but also to highlight the importance of early detection either by prenatal imaging or careful evaluation of differential diagnosis of common symptoms. We herein, describe two infants with large cardiac tumours (fibroma and teratoma) both arising from the interventricular septum and underwent surgical excision. A possible role of cardiac remodeling in myocardial tissue healing after extensive tissue resection in such patients is hypothesised through available experimental or limited clinical information.

## Background

Primary cardiac tumours in paediatric population are an unusual occurrence with reported incidence of 0.17-0.28% as per echocardiographic or autopsy series [1,2]. Although, majority of such tumours are benign (90%), the frequency and type of cardiac tumours in this age group is different from the adult population [2]. Rhabdomyoma is the most common cardiac tumour in children, representing more than 60% of primary tumours, followed by teratoma, fibroma and haemangioma [1-3]. Unlike adults, myxoma is rarely seen in children [1-3]. Echocardiography, Computerized Tomography (CT) and Magnetic Resonance Imaging (MRI) of the heart are the non-invasive diagnostic tools but histopathology examination remains the conclusive evidence [1-3].

There are several consecutive series published in the last decade on cardiac neoplasms [1-5]. However, this report is not only an effort to contribute to the existing literature for better understanding and management of similar patients but also to highlight the importance of early detection by a careful consideration of differential diagnosis of common cardiac symptoms or better use of prenatal imaging techniques for better outcomes. Hypothetically, in paediatric patients, myocardial tissue remodeling may play a role in healing or recovery of cardiac functions in long-term especially in patients who suffer extensive tissue loss during tumour resection. However, this concept is still not fully understood and the current knowledge is based on either experimental data or limited clinical studies in adult population with ischemic heart disease and cardiac failure [6]. Therefore, we have tried to highlight the possible mechanism and role of tissue remodeling in this scenario which could makes children not only tolerant to extensive myocardial tissue loss but also helps in recovery in long term. We herein, describe two infants with cardiac tumours (fibroma and teratoma) both arising from the interventricular septum and underwent surgical excision.

## Case presentation

### Patient-1

**Figure 1:**
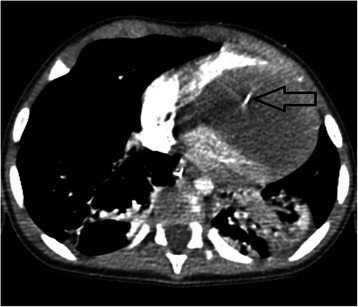
**Computerized scan of the chest with contrast showing a well-defined large (4×4 cm) space occupying lesion with near-fluid density filling the ventricular cavities attached to the interventricular septum and displacement of surrounding structures.** Mild calcification at the centre is noted (arrow).

**Figure 2:**
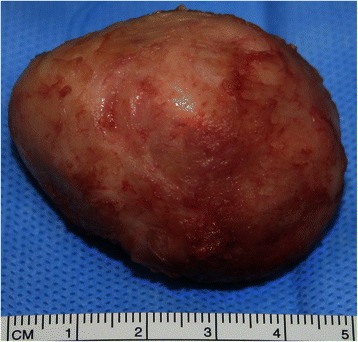
**Excised tumour showing grey white mass (4×4 cm) with hard consistency.**

**Figure 3:**
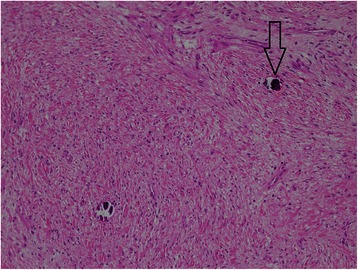
**A well-demarcated spindle cell lesion with fibromyxoid and vascular stroma.** Rare micro-calcifications are also noted (arrow) (hematoxylin and eosin stained, 10x).

A 12-month-old, Arabic, patient was admitted on emergent basis with multifocal ventricular ectopic beats associated with low cardiac output. He was intubated and resuscitated. Echocardiography demonstrated a large tumour mass filling the apical parts of both the ventricular cavities with severe restriction to the diastolic filling. Other cardiac structures were normal. The CT angiography of the chest revealed a well-defined, near-fluid density, large, space-occupying lesion within the heart involving the anterior-inferior part of the interventricular septum bulging into the ventricular cavity and compromising it (Figure 1). There were some cystic changes and patchy calcifications in the tumour. Emergent cardiac surgery was performed through the median sternotomy under standard cardiopulmonary bypass (CPB) using aortic and bicaval cannulation at moderate systemic hypothermia. Under cardioplegic arrest and aortic cross clamping, the right ventricle was opened parallel to the left anterior descending artery. Resection of the mass (4×4 cm) was done “in-toto” including the part of ventricular septum (Figure 2). The resultant thinning of the septum was reinforced with a 0.4mm polytetrafluoroethylene patch (PTFE). The PTFE was preferred due to low thrombogenicity, less porosity and biostability. The patient was weaned from the CPB and a postoperative transoesophageal echocardiography confirmed the removal of tumour mass and absence of residual shunts within the cardiac chambers. The histopathological examination of the excised tumour confirmed the diagnosis of a well encapsulated matured fibroma (Figure 3). The patient was discharged home and at 1 year follow up he was asymptomatic with no evidence of recurrence on echocardiography.

### Patient-2

**Figure 4:**
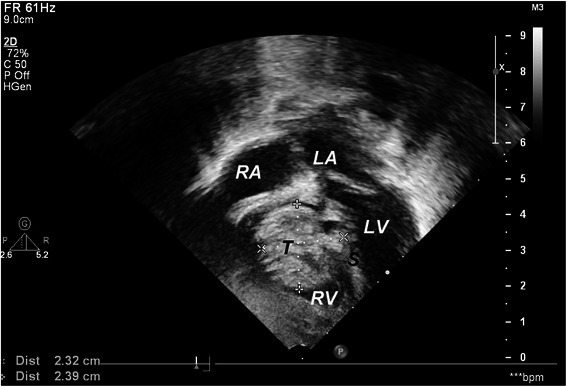
**Two-Dimensional echocardiogram (transthoracic) showing well-defined tumour mass (T) attached to the interventricular septum (S) adjacent to the tricuspid valve on the right ventricle cavity.** (LV-Left Ventricular Cavity, RA-Right Atrium, LA-Left Atrium, RV-Right Ventricular Cavity).

A 1–month-old, Arabic, female patient was referred for evaluation of a cardiac murmur due to suspected cardiac tumour. The patient was asymptomatic and clinically the general and physical examination was un-remarkable except that there was a faint precordial systolic murmur. Alfa Feto Protein (AFP) was elevated (118 IU/ml). Echocardiogram revealed a cystic mass (2.5×2.5 cm) attached to the interventricular septum near the tricuspid valve on the right ventricular cavity without any obstruction or compromise to the flow (Figure 4). Other cardiac structures were normal without evidence of infectious or thrombotic origin of the mass.

**Figure 5:**
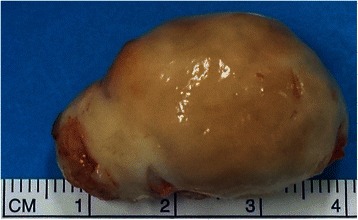
**Excised tumour (4×3 cm) showing nodular grey white mass with variable consistency.**

**Figure 6:**
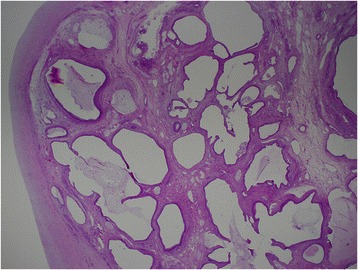
**A well-demarcated mass showing different mature tissues and dilated cystic spaces lined by respiratory- and mucinous-type epithelium.** No immature elements of germinal tissue noted (hematoxylin and eosin stained, 10x).

The patient underwent surgical excision of the tumour under standard CPB, bicaval and aortic cannulation, aortic cross-clamping and cardioplegic arrest through the right atrial approach at moderate systemic hypothermia. A large tumour mass (4×3 cm) originated from the interventricular septum and filled the ventricular cavity, adjacent to the tricuspid valve (Figure 5). With careful dissection, the tumour was fully resected without injury to the tricuspid valve or creating defect. The patient was weaned from the CPB and was stable throughout. The histopathology confirmed teratoma featuring a derivative from all three germ layers. There were focal cystic, degenerative and micro-calcific changes seen in the well demarcated excised mass with a thick fibrous capsule. The dilated cystic spaces were lined by respiratory- and mucinous-type epithelium (Figure 6). Postoperative course was uneventful. The follow up at 6 months was unremarkable except the AFP is down to 58 IU/ml.

## Discussion

Fibroma accounts for 12-16% of primary cardiac tumours in children and usually derived from connective tissue fibroblasts [3-6]. Cardiac fibromas are mainly located in the ventricles and attached to the interventricular septum. They can interdigitate with ventricular muscle at the tumour border and replace functioning muscle mass which may result in intractable congestive heart failure [3,4]. Interior calcification has been described as pathognomonic for fibroma and reflects poor blood supply to the mass [3,4]. Arrhythmias are not rare and have been described in many patients with cardiac fibromas. This is due to the fact that fibromatous tissue may expand into the ventricular conduction tissue. Our patient had ventricular ectopic that resolved after tumour excision. These tumours typically remain dormant but spontaneous regression rarely occurs.

Cardiac teraromas are the second most common tumour in the neonates [3-5]. The usual occurrence is in the pericardial cavity attached to the aorta or pulmonary artery. Intracardiac teratoma arises from the atrial or ventricular wall as a nodular mass and produce obstructive features. Although teratomas have been considered to be benign, tumour recurrence after resection or even malignant transformation may be a possibility. Close follow-up is necessary to avoid a delay in diagnosis. Typically a teratoma consists of derivatives from all 3 germ layers and may have mature or immature tissues.

In infants and children, tumour presentation is often variable due to its location and size. Cardiac tumours are frequently hemodynamically relevant due to obstructive features at various locations within the heart. It includes obstruction to the ventricular inflow and outflow tracts, coronary arteries or the valves. Even ventricular arrhythmias may be the presenting features. Furthermore, tumour size matters in two different ways. Either tumour mass obstructs flow within cardiac chambers that may be well-tolerated for a long period or tumour encroaches on the wall of surrounding structures and presents with arrthythmia or cardiogenic shock (patient 1). Our second patient had a relatively-late presentation because some time must have been taken to grow an intramural lesion to attain 4 cm in diameter.

Echocardiography is the mainstay of non-invasive diagnostic tool that depicts tumour size, location, surrounding structures and any functional impairment. Cardiac catheterization is usually not necessary except when there are large tumour compromising coronary arteries or associated pathologies.

Supplemental diagnostic imaging may include CT or MRI scanning because it provides sectional views of cardiac and mediastinal structures from various angles. In addition, it delineates tissue composition to identify solid, liquid, fatty, haemorrhagic, calcific or cystic nature of the tissue [5]. Currently, the improvement in imaging technologies not only allow the diagnosis of cardiac tumours as early as during fetal life but also help in planning surgery or follow up due to their quality of images [5].

It is always doubtful to ascertain which patients need surgery and which will benefit from conservative follow-up [4]. Surgical intervention is warranted for symptomatic patients [4]. In our first patient, hemodynamic compromise and ventricular ectopic were indications for emergent surgical intervention. However, asymptomatic patients with cardiac tumours may only need a careful long-term follow up or surgical considerations as a preventive measure to avoid complications [4]. Although most of the paediatric cardiac tumours are benign and rare, there may be significant morbidity or mortality. This fact should be considered in selecting asymptomatic patients for surgery. Despite our second patient was asymptomatic, surgery was considered due to tumour’s size and to prevent thromboembolism or damage to adjacent structures (i.e. tricuspid valve). The aim of surgical management of cardiac tumours is to achieve total resection of the mass, if possible and most importantly restore the hemodynamic function of the heart [4].

In symptomatic patients, if the resection of the cardiac tumour is not achievable, or a large resultant structural cardiac defect is anticipated, cardiac transplantation remains a possibility where there is no evidence of metastatic involvement. However, long-term results are questionable [3].

When tumour is encapsulated it keeps the integrity of the myocardial tissue without destroying or infiltrating them. Thus, after tumour removal relatively intact muscular layers can restore geometry and function favourably. This is similar mechanism we observe after intraluminal mass removal, i.e. cavity dilation goes back to normal. However, majority of the large cardiac tumours in paediatric age group are associated with structural change in the myocardial tissue either due to destruction or infiltration effect. In addition, surgical resection causes further damage or tissue loss. This fact prompts us to think about the mechanism of repair and favourable outcome in paediatric population with cardiac tumours. In our opinion, cardiac remodeling plays an important role in restoration of size, shape and function of the myocardial tissue and the heart in such patients. Although patients with major remodeling demonstrate progressive worsening of cardiac function, slowing or reverse remodeling has recently been a goal of heart failure therapy world-wide [7]. In experimental models the process of ventricular remodeling begins within first few hours and continue to progress in long-term [7]. The myocyte, interstitium, fibroblasts, collagen and coronary vasculature are the main component of cardiac remodeling. Cardiac remodeling has been described as both adaptive and a maladaptive process, with adaptive component enabling the heart to maintain function in acute phase of cardiac injuries. Few clinical trials and experimental studies in adults have confirmed therapeutic approaches, such as angiotensin converting enzymes inhibition and beta-blockage, which may reduce morbidity and mortality or in some cases improve a number of remodeling parameters or enhance reverse remodeling [7-9]. This fact may have a bearing in management of patients with large cardiac tumours before or after surgery.

## Conclusions

Cardiac tumours in paediatric population must be considered in the differential diagnosis of cardiac insufficiency, valvular disease, arrhythmias, cardiomegaly or presence of murmurs. The prenatal screening should use appropriate imaging tools for early detection of such tumours. Surgical consideration should include invidualised, early and safe management approach for symptomatic patients aiming not only to resect the entire tumour mass with sufficient margin but more importantly restore best possible haemodynamics with a careful follow up. The role of cardiac remodeling and pharmacological intervention to alter this process may have future implications especially to heal damaged myocardial tissues secondary to large size of tumours or surgery.

## Consent

Written informed consent was obtained from the patient for publication of this Case report and any accompanying images. A copy of the written consent is available for review by the Editor-in-Chief of this journal.

## Abbreviations

CT: Computerised tomography
MRI: Magnetic resonance imaging
CPB: Cardiopulmonary bypass
PTFE: Polytetrafluoroehylene
AFP: Alfa feto protein
T: Tumour mass
S: Interventricular septum
LV: Left ventricular cavity
RA: Right atrium
LA: Left atrium
RV: Right ventricular cavity
